# The combined effects of *Map3k1* mutation and dioxin on differentiation of keratinocytes derived from mouse embryonic stem cells

**DOI:** 10.1038/s41598-022-15760-z

**Published:** 2022-07-07

**Authors:** Jingjing Wang, Bo Xiao, Eiki Kimura, Maureen Mongan, Ying Xia

**Affiliations:** Department of Environmental and Public Health Sciences, College of Medicine, University of Cincinnati, Cincinnati, OH 45267-0056 USA

**Keywords:** Biological techniques, Cell biology, Developmental biology, Stem cells, Environmental sciences

## Abstract

Epithelial development starts with stem cell commitment to ectoderm followed by differentiation to the basal keratinocytes. The basal keratinocytes, first committed in embryogenesis, constitute the basal layer of the epidermis. They have robust proliferation and differentiation potential and are responsible for epidermal expansion, maintenance and regeneration. We generated basal epithelial cells in vitro through differentiation of mouse embryonic stem cells (mESCs). Early on in differentiation, the expression of stem cell markers, *Oct4* and *Nanog,* decreased sharply along with increased ectoderm marker *keratin (Krt) 18*. Later on, *Krt 18* expression was subdued when cells displayed basal keratinocyte characteristics, including regular polygonal shape, adherent and tight junctions and *Krt 14* expression. These cells additionally expressed abundant Sca-1, Krt15 and p63, suggesting epidermal progenitor characteristics. Using *Map3k1* mutant mESCs and environmental dioxin, we examined the gene and environment effects on differentiation. Neither *Map3k1* mutation nor dioxin altered mESC differentiation to ectoderm and basal keratinocytes, but they, individually and in combination, potentiated *Krt 1* expression and basal to spinous differentiation. Similar gene-environment effects were observed in vivo where dioxin exposure increased Krt 1 more substantially in the epithelium of *Map3k1*^+*/-*^ than wild type embryos. Thus, the in vitro model of epithelial differentiation can be used to investigate the effects of genetic and environmental factors on epidermal development.

## Introduction

The development of the epidermis is a consecutive multi-step process that commences at the differentiation of stem cell to surface ectoderm, which in turn differentiates to the keratinocytes^1,2^. Keratinocytes are the principal constitutes of the epidermis, composed of four layers, basal, spinous, granular and cornified envelop. Cells in each layer have distinct morphological and biochemical properties and gene expression^3^. Typically, the basal keratinocytes express keratins (Krt) 5 and Krt 14, the spinous keratinocytes express Krt 1 and Krt 10, and the granular and cornified keratinocytes express Involucrin, Loricrin and Filaggrin^4^. Only the basal keratinocytes are capable of proliferation, and they serve as the reservoir to supply and replenish cells in the suprabasal layers.

Disruption of the differentiation program of the epidermis leads to developmental defects^5–9^. Skin abnormalities, such as neurofibromatosis, xeroderma pigmentosum, epidermolysis bullosa, and the most common psoriasis and atopic eczema, are costly congenital disorders that often require lifetime health care^10^. Mutations of genes associated with epithelial differentiation account for some anomaly cases, whereas environmental factors acting through epigenetic modulations are also known to be an etiology for the diseases^11–14^. Up to date, the genetic and environmental etiology for many skin disorders has remained poorly understood.

MAP3K1, also known as MEK Kinase 1 (MEKK1), is a member of the MAP3K superfamily, and plays highly specific roles in signal transduction and embryonic development^15^. MAP3K1 deficient mice have a birth defect of the eye, due to abnormal epithelial morphogenesis in the embryonic eyelids^16^. While how MAP3K1 affects epithelial morphogenesis is still under investigation, the gene expression signatures in epithelial cells isolated from wild type and *Map3k1* knockout mice show that MAP3K1 may impede basal to suprabasal differentiation^17^. This role of MAP3K1, however, had neither been validated nor further explored.

The dioxin-like chemicals (DLCs) represent a large group of chemicals that are wide-spread environmental contaminants^18^. The DLCs are generated either naturally through processes like forest fires and volcanic eruptions or by industrial activities, such as incomplete combustions. These chemicals are stable in the environment with a half-life of several years. Therefore, human exposure is inevitable. DLC exposure has been linked to many developmental defects^19–22^. Using 2,3,7,8-Tetrachlorodibenzo-p-dioxin (TCDD) as a model DLC congener, studies in laboratory rodents show that in utero dioxin exposure causes diverse developmental abnormalities, including, but not limited to, hydronephrosis, cleft palate, and vaginal thread formation^23^. In the epidermis, dioxin is shown to accelerate terminal differentiation, leading to acanthosis and epidermal hyperkeratosis phenotypes in mice and potentiate terminal differentiation of human keratinocytes in vitro^24,25^. Moreover, dioxin enhances differentiation of cells that have already committed to differentiation, insinuating that this environmental toxicant affects differentiation in a developmental stage-specific manner^26^.

Mouse embryonic stem cells (mESCs) are the inner cell mass cells isolated from the pre-implantation blastocysts^27,28^. When maintained under a well-defined culture condition, the mESCs have unlimited capacity of self-renewal, remain pluripotent with abundant expression of the pluripotency genes, such as *Oct3/4 (Pou5f1)* and *Nanog*^29^. The mESCs also have potent potential of differentiation to generate all the cell types of the body^30^. Under defined inducing conditions, they produce cells of the three primary germ layers, i.e. mesoderm, definitive endoderm and ectoderm, which in turn give rise to progenitor and mature cells of various lineages. Differentiation of mESC to generate epithelial cells for the purpose of tissue engineering and wound healing has been reported^31^, but it has not been used to explore the genetic and/or environmental factors as the underlying etiology of epithelial disorders.

In this study, we differentiated mESCs to basal keratinocytes in vitro. Retinoic acid (RA) and bone-morphogenetic protein-4 (BMP4) were used to induce early stage commitment of the surface ectodermal lineages; Defined keratinocyte serum free medium (DKSFM) were used to drive further differentiation to and the expansion of basal keratinocytes. The resultant basal keratinocytes, with epidermal progenitor signatures, could be passaged at least 20 times with minimal terminal differentiation. Using this system, we investigated the effects of the environmental toxicant dioxin and *Map3k1* gene mutations, either individually or in combination, on epidermal differentiation. The results highlight the utility of the in vitro system to investigate risk factors and multifactorial etiology in congenital skin disorders without extensive utilization of live animals.

## Results

### In vitro differentiation of mouse ESCs to keratinocyte

**Figure 1:**
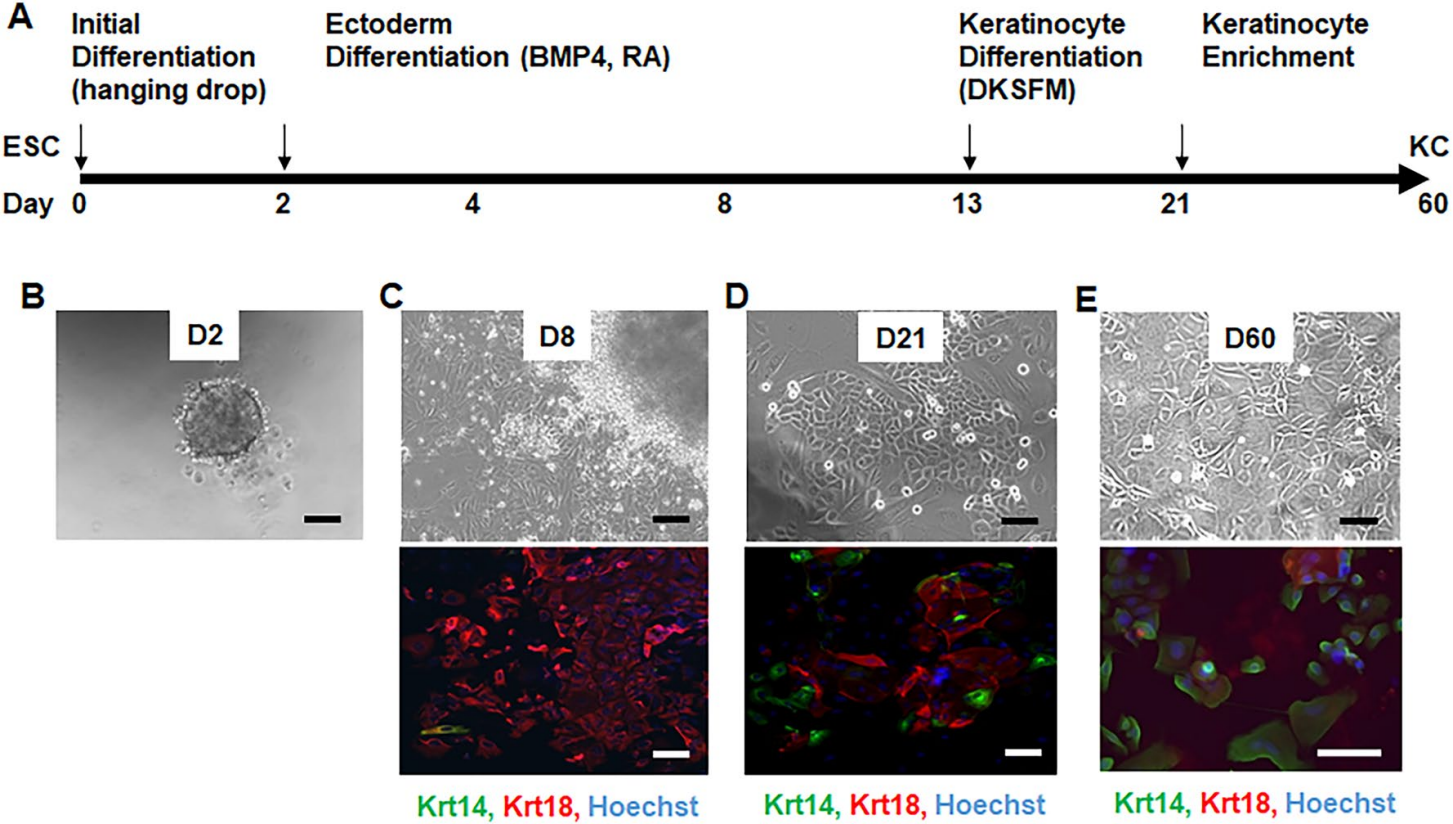
Differentiation of mESCs to keratinocytes in vitro. (**A**) Scheme of in vitro mESC to keratinocyte differentiation. Representative brightfield and immunofluorescent staining images of (**B**) cell aggregates and EBs on Day 2 (D2), (**C**) attached EBs grown in presence of RA and BMP4 for 6 days (D8) with cells outgrown from the EBs expressing Krt 18 and few cells at the most outer layer started to express Krt 14, (**D**) Passaged cells at day 21 of differentiation formed colonies with more Krt 14 positive cells, and (**E**) Majority cells became Krt 14 positive after multiple passages and culture for 1–2 months (D60). Scale bar represents 200 μm.

We differentiated the mESCs in vitro using a protocol adapted from Bilousova et. al. and Metallo et al. with modifications to increase efficiency at the initial phase of the procedure^32,33^ (Fig. 1A). Specifically, mESCs were re-suspended in EB media and the hanging drop methods were used for EB formation. Under these conditions, compact, sphere-shaped EBs formed in nearly 80% of the handing drops (Fig. 1B). After plating on ColIV-coated plates and growing in DKSFM media, the EBs had many cells growing out from the center (Fig. 1C). The outgrown cells displayed predominant ectodermal characteristics with strong expression of the surface ectodermal marker Krt 18. After passage and culture for 21–60 days, the cells started to exhibit basal keratinocyte-like morphology with increased expression of the basal keratinocyte marker Krt 14 (Fig. 1D and E). At proximately passage 6 (P6), the cells began to express abundant Krt 14 that was sustained subsequently in at least 20 passages. The cells can also be stored in and recovered from liquid N_2_ with no apparent change of basal keratinocyte morphology and Krt 14 expression.

**Figure 2:**
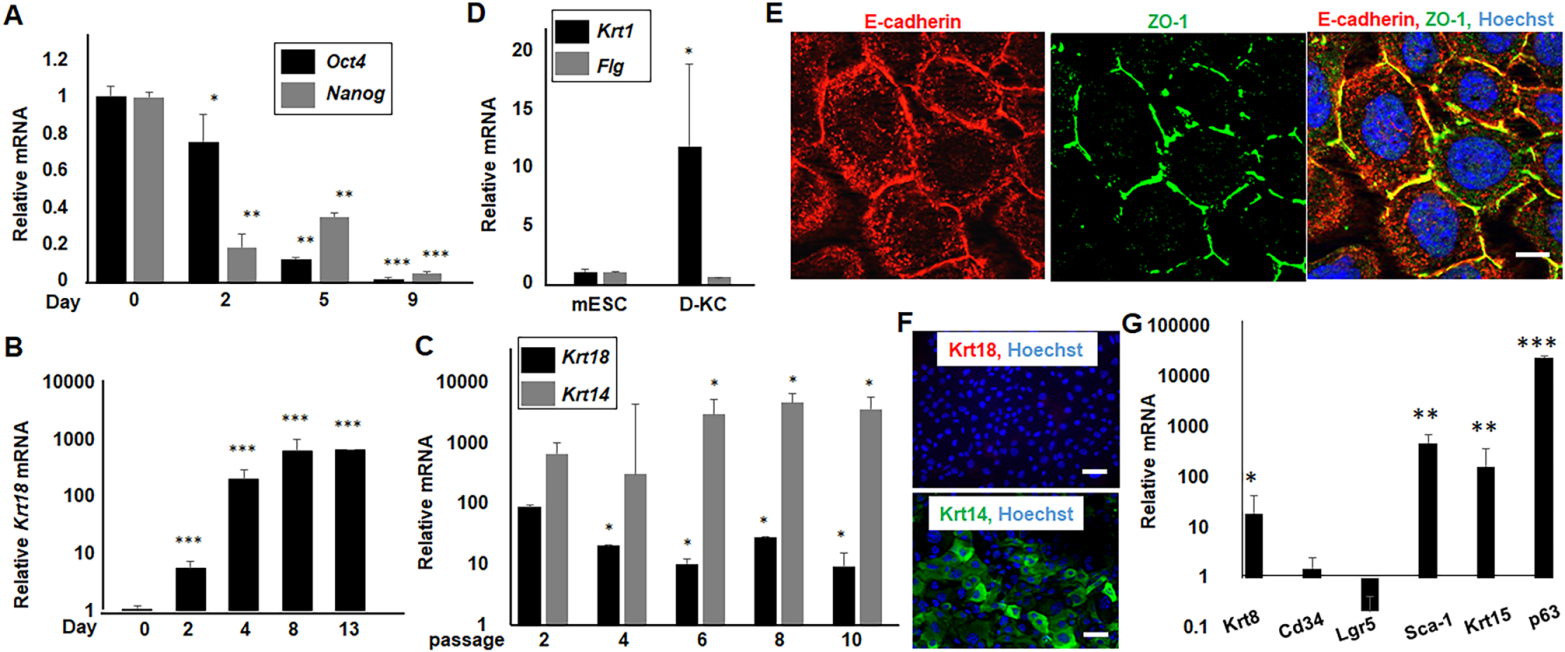
Molecular tracing of the in vitro differentiated cells. (**A**, **B**) Cells at different days of differentiation were examined for gene expression by RT-PCR. The expression of (**A**) the stem cell markers *Oct4* and *Nanog,* and (**B**) the ectoderm marker *Krt 18* were compared to the levels in undifferentiated mESCs, i.e. day 0. (**C**) Cells at different passages were examined for mRNA of *Krt 18* and *Krt 14.* The expression of *Krt 18* was significantly decreased while the expression of the basal keratinocyte marker *Krt 14* was significantly increased with the increase of passages. High level *Krt 14* expression was stabilized at passage 6 onward. These cells are so forth termed differentiated-keratinocyte (D-KC). Compared to mRNA levels in mESCs, the D-KC had (**D**) elevated expression of the spinous cell marker *Krt 1,* but not the cornified envelop marker *Flg*, and (**G**) a slight increased *Krt 8*, no change of hair follicle stem cell markers, *Cd34* and *Lgr5*, and significant increase of the epidermal progenitor markers *Sca-1*, *Krt15* and *p63*. D-KC were subjected to immunofluorescence staining for (**E**) the adherence junction (E-cadherin) and tight junction (ZO-1), and (**F**) anti-Krt 18 and anti-Krt 14. nucleus was stained with Hoechst 33,342 (blue). *p < 0.05, **p < 0.01, ***p < 0.001 were considered statistically significant compared to day 0 or passage 2. Scale bar represents 20 μm.

Examination of the cells at mRNA level at different days of in vitro culture revealed a gradual decrease of the expression of stem cell markers, *Oct4* and *Nanog*, which became nearly undetectable by day 9. Conversely, there was a significant increase in the expression of *Krt 18* (Fig. 2A and B). The *Krt 18* expression reached the peak levels at 8–13 days of differentiation, but decreased sharply afterwards, and remained at a steady low level at the 4th passage and onwards. Concurrently, the *Krt 14* expression increased gradually and arrived at a steady high level after a few passages (Fig. 2C). While these cells had a slight increased expression of suprabasal gene, *Krt 1*, they had no detectable *Filaggrin* (*Flg*) that are expressed only in the uppermost layer of the epidermis at late stage of fetal development (Fig. 2D). The adherence junctions and tight-junctions, key characteristics of the basal keratinocytes detected by E-cadherin and ZO-1 staining, were evident at the intercellular contacts (Fig. 2E)^34,35^. These cells maintained a high level of Krt 14 expression in > 90% cells with no detectable Krt18 expression (Fig. 2F) for > 20 passages, including after recovery from storage in liquid N2, and are henceforth termed differentiated-keratinocyte (D-KC).

The continuous passaging capacity led us to examine whether the D-KC possessed stem and/or progenitor characteristics. The D-KC exhibited stable low expression of *Krt 8*, which, like Krt18, is a surface ectoderm marker (Fig. 2G). On the other hand, while negative for *Cd34* and *Lgr5*, putative markers for hair follicle bulge stem cells^36,37^, the D-KC had abundant expression of *Sca-1*, *Krt 15* and *p63,* which are markers for epidermal progenitors^38–40^. Thus, the D-KC seem to possess progenitor properties, empowering the continuous growth and subculture capacities.

### The role of MAP3K1 in keratinocyte differentiation

**Figure 3:**
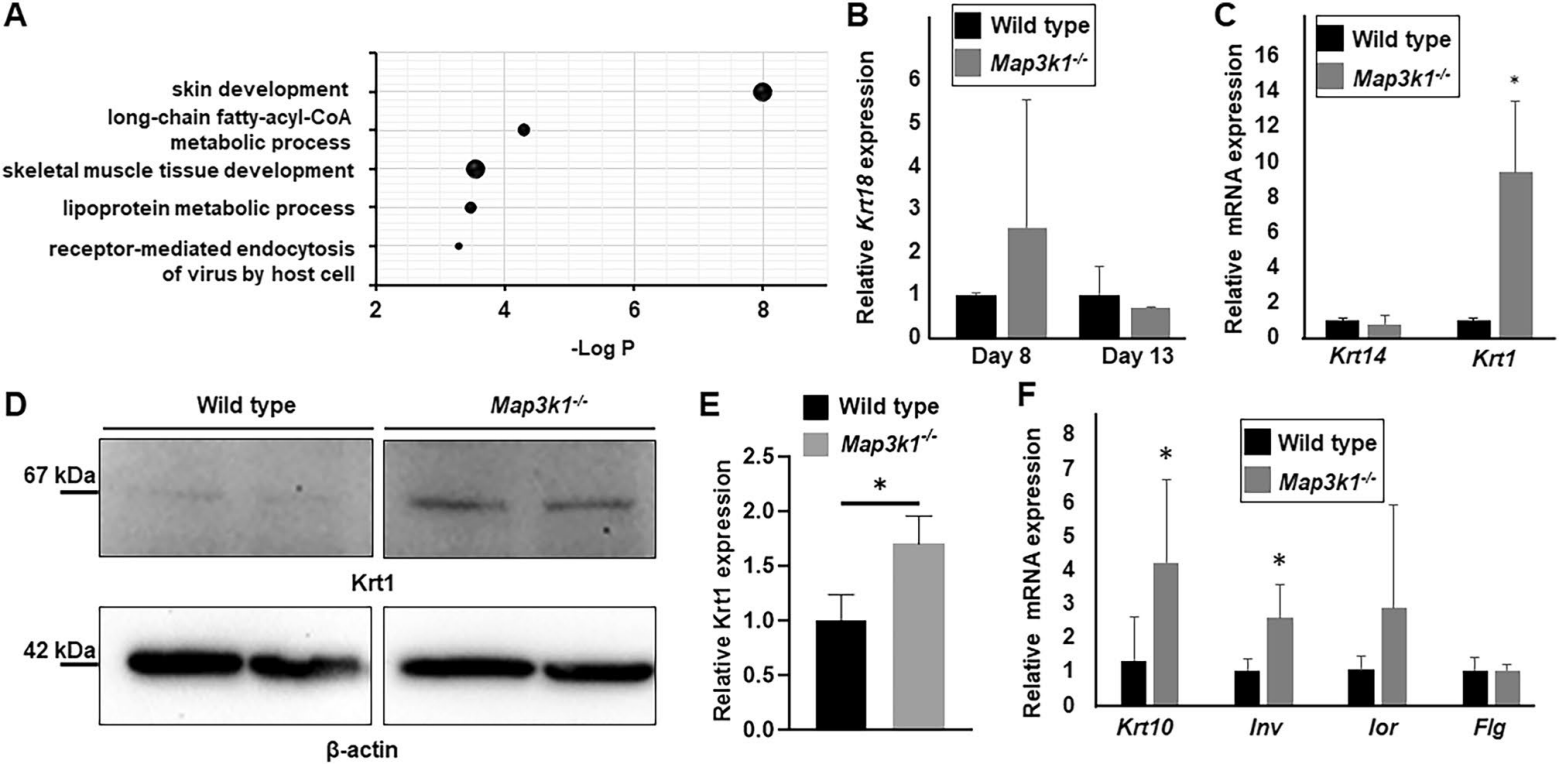
MAP3K1 loss-of-function enhanced suprabasal differentiation. (**A**) Differentially expressed genes in primary keratinocytes isolated from wild type and *Map3k1*^*-/-*^ mice were subjected to GO pathway analyses and the top biological functions up-regulated in the *Map3k1*^*-/-*^ versus wild type cells were shown. The wild type and *Map3k1*^*-/-*^ mESCs were differentiated in vitro for various times and examined for mRNA of (**B**) *Krt 18* at the early phases, and (**C**) *Krt 14* and *Krt 1* and (**F**) *Krt 10, Inv, Lor, and Flg*, at the late phases (after 6 passages). The wild type and *Map3k1*^*-/-*^ D-KC were subjected to Western blotting using antibodies indicated (**D**) and results of at least 4 data sets were quantified (**E**). Original immunoblots corresponding to (**D** and **E**) are presented in Supplementary Fig. S1. The expression in *Map3k1*^*-/-*^ were compared to those in wild type cells, set as 1. *p < 0.05 is considered statistically significant.

MAP3K1 is a signal transduction enzyme playing key roles in embryonic development and epithelial morphogenesis^16^. Although the *Map3k1*^*-/-*^ mice do not have overt skin defects, they display eye developmental defects due to abnormal epithelial morphogenesis and delayed healing of skin full-thickness wounds^17^. To explore the role of MAP3K1 in skin biology, we re-analyzed the global gene expression in wild type and *Map3k1*^*-/-*^ primary keratinocytes^17^, using a more stringent cut-off criteria and performed GO analyses of the differentially expressed genes. We found that skin development was a top biological process affected by MAP3K1 (Fig. 3A). Specifically, genes in epithelial terminal differentiation were significantly up-regulated in *Map3k1*^*-/-*^ versus wild type cells^17^ (Supplementary Table s1).

To evaluate whether the roles of MAP3K1 in epithelial differentiation could be recapitulated in the in vitro differentiation model, we differentiated the wild type and *Map3k1*^*-/-*^ mESCs and examined the expression of marker genes at different time intervals. Wild type and *Map3k1*^*-/-*^cells had similar expression of *Krt 18* at the early phase of differentiation (Fig. 3B). While these cells also had similar *Krt 14* expression, they were strikingly different on *Krt 1* expression at the later phase of differentiation (Fig. 3C). The expression levels of *Krt 1* mRNA were tenfold more abundant in *Map3k1*^*-/-*^ than in wild type cells. Similarly, there was a significant nearly twofold higher Krt 1 protein in *Map3k1*^*-/-*^ than wild type cells (Fig. 3D and E). To validate the differentiation status of the D-KC cells, we examined additional markers. Compared to the wild type cells, the *Map3k1* knockout cells had a slightly higher expression of *Krt 10* and *Involucrin*, markers for spinous and granular cells, respectively; however, they had similar expression of *Loricrin* and *Filaggrin*, which are markers for the most outer layer stratum corneum (Fig. 3F)^41^. The in vitro data validate that MAP3K1 hampers epithelial differentiation, an idea originally insinuated from global gene expression studies.

### Dioxin potentiate keratinocyte differentiation in vitro

**Figure 4:**
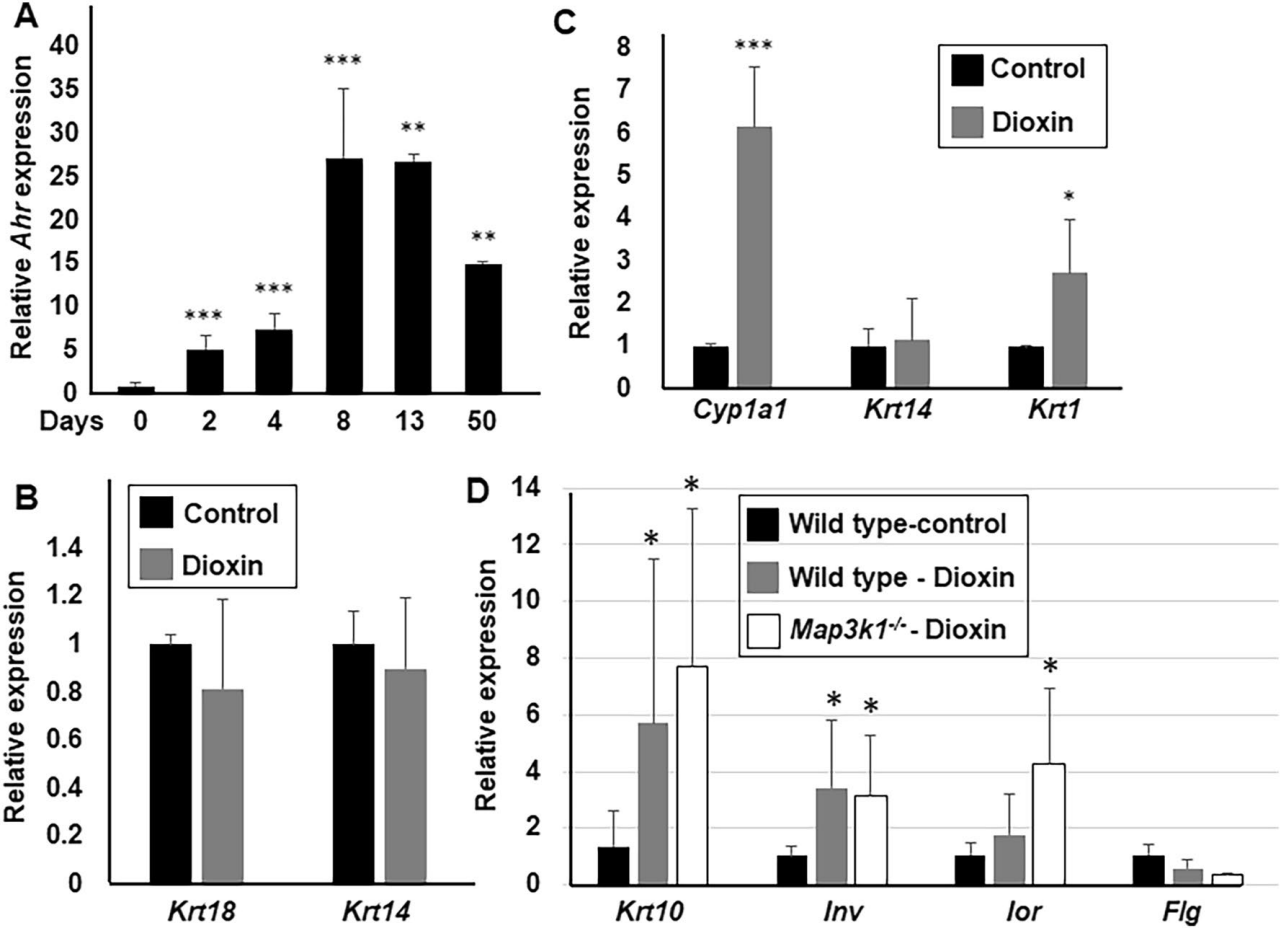
Dioxin potentiated suprabasal differentiation. Gene expression was examined using RT-PCR. (**A**) The *Ahr* expression was compared to levels in undifferentiated mESCs, set as 1. The expression of *Krt 18*, *Krt 14*, *Krt 1, Krt 10, Inv, Lor, Flg* and the AHR activation marker, *Cyp1a1* were examined (**B**) in cells differentiated for 13 days and (**C** and **D**) in wild type and *Map3k1*^*-/-*^ D-KC, in the presence and absence of 10 nM dioxin as indicated. Statistical analyses and relative expression are based on comparison to (**A**) undifferentiated mESCs and (**B-D**) control wild type samples, set as 1. *p < 0.05, **p < 0.01 and ***p < 0.001 are considered significant.

The global environmental pollutant dioxin exhibits diverse developmental toxicities and is suggested to cause acanthosis and epidermal hyperkeratosis through derailing epithelial differentiation^24^. As most dioxin effects are mediated by the Aryl Hydrocarbon Receptor (AHR), a ligand-activated transcription factor that regulate dioxin-responsive genes^42^, we examined AHR expression during in vitro epithelial differentiation. The expression of *Ahr* was negligible in mESCs, but was significantly increased as soon as the cells started to differentiate in the EBs at day 2 of differentiation, consistent with previous observations^43^ (Fig. 4A). The *Ahr* expression continuously increased and remained at high levels after the cells committed to primary ectodermal lineages and became basal keratinocytes, suggesting that AHR signaling could be activated by dioxin as soon as the cells exit stemness.

To evaluate the effects of dioxin on differentiation, we examined cells differentiated in media with or without dioxin for 13 days. The presence of dioxin in the culture media did not alter the expression of *Krt 18* and *Krt 14*, suggesting that dioxin did not change the course of differentiation from mESC to surface ectoderm and basal keratinocytes (Fig. 4B). After multiple passages (> 8), the steady-state D-KC exhibited a robust dioxin-induced AHR activation, reflected by the induction of *Cyp1a1*, the prototypical AHR target gene (Fig. 4C). While the presence of dioxin did not change *Krt 14* expression, it increased *Krt 1* expression by threefold, although such increase was not detected at the protein level likely due to insufficient sensitivity of the detection methods (data not shown). In addition to *Krt 1*, *Krt 10* and *Involucrin* mRNA were also slightly induced by dioxin treatment (Fig. 4D), supporting that dioxin treatment potentiates basal to spinous keratinocyte differentiation.

### Dioxin plus Map3k1 loss-of-function further promote differentiation

**Figure 5:**
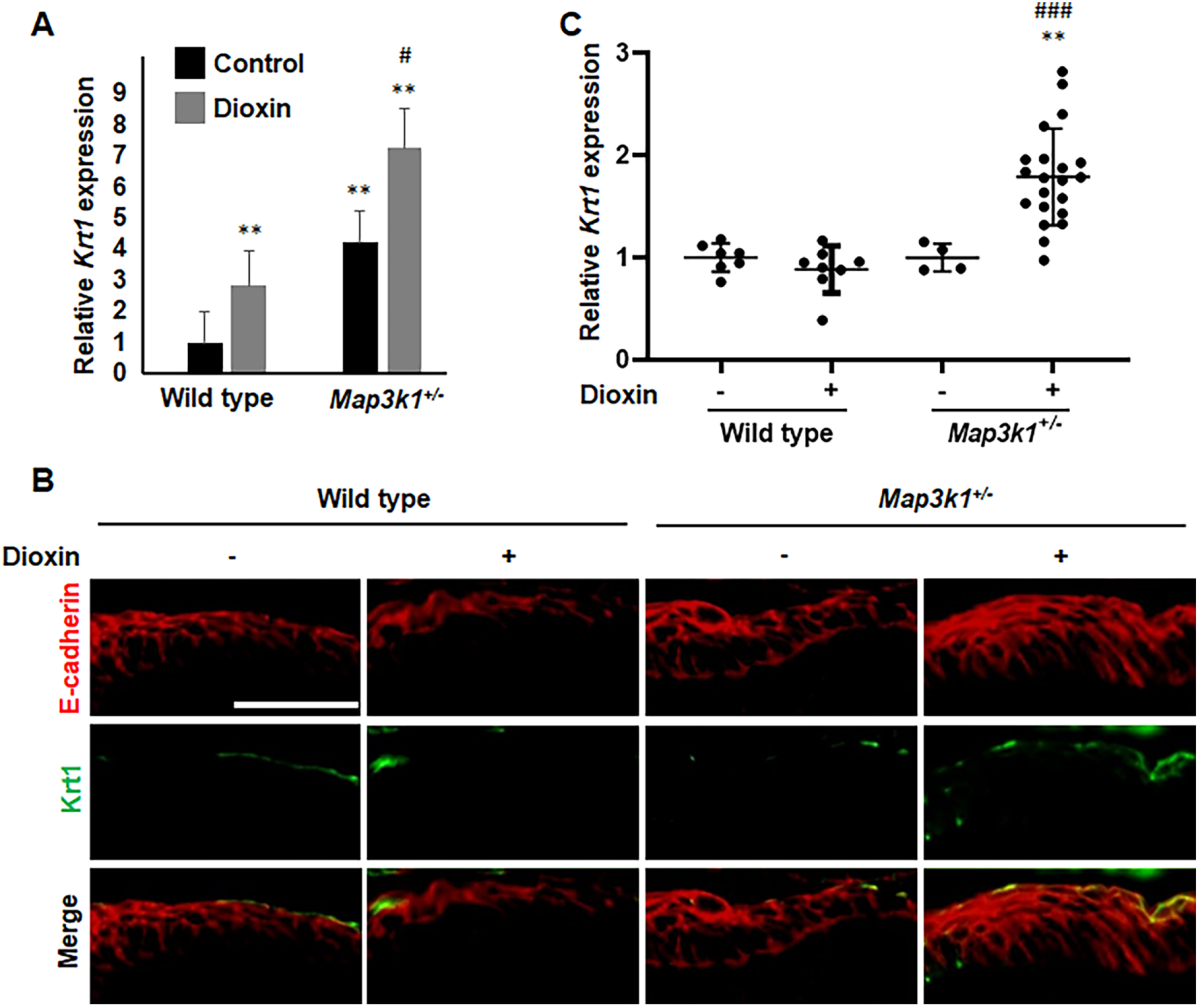
Dioxin plus *Map3k1* loss-of-function potentiated keratinocyte terminal differentiation. The *Krt 1* expression was examined **(A)** by RT-PCR in wild type and *Map3k1*^+*/-*^ D-KC with or without 10 nM dioxin treatment for 3 days, and (**B**) by immunohistochemistry in wild type and *Map3k1*^+*/-*^ E15.5 embryos with or without 50 ug/kg dioxin treatment at E11.5; E-cadherin labeled all epithelial cells. (**C**) Quantification of Krt 1-positive signals in (**B**). **p < 0.01 are considered significantly different compared to wild type untreated samples, and ^#^*p* < 0.05, ^###^*p* < 0.001 are considered significantly different compared to untreated samples of the same genotype. Scale bar represents 50 μm.

In vivo, dioxin and *Map3k1*^+*/-*^ have synergistic effects on impairing eye development. When neither dioxin nor *Map3k1*^+*/-*^ alone are detrimental, their combination causes birth defects of the eye, a defect observed also in un-treated *Map3k1*^*-/-*^ mice^44^. Notwithstanding the intriguing phenotypic observations, how the environmental and genetic factors converge to disrupt the developmental programs has remained elusive. Given the similar effects of dioxin and *Map3k1* gene mutation on promoting suprabasal differentiation, we postulated that the combination of these conditions exacerbated the differentiation abnormalities. In supporting of this idea, we noted that compared to the un-treated and dioxin-treated wild type cells, the dioxin-treated *Map3k1*^*-/-*^ cells had also increased expression of *Loricrin*, another cornified envelop marker (Fig. 4D). We additionally tested the idea by treatment of D-KC derived from wild type and *Map3k1*^+*/-*^ mESCs with 10 nM dioxin for 3 days and examination of *Krt 1* expression. Compared to the wild type cells, the *Map3k1*^+*/-*^ cells had higher *Krt 1* expression (Fig. 5A). Dioxin treatment increased *Krt 1* expression by threefold in wild type cells, and remarkably, it induced *Krt1* expression further in *Map3k1*^+*/-*^ cells. The *Krt 1* expression in dioxin-treated *Map3k1*^+*/-*^ cells reached to nearly eightfold of the levels in the untreated wild type cells and close to the levels in the untreated *Map3k1*^*-/-*^ cells (Figs. 3C and 5A).

We also tested this idea in vivo by treatment of pregnant mice, carrying wild type and *Map3k1*^+*/-*^ embryos, with 50 ug/kg dioxin on embryonic day (E)11.5. The embryos were collected on E15.5, as described previously^44^ and the embryonic skin was examined by immunohistochemistry. The E-cadherin staining labeled multiple layers of the epithelial cells in the embryonic skin, in which the Krt 1 positive cells were detectable at the most outer layer (Fig. 5B). Quantification of the signal intensities showed that neither dioxin exposure nor *Map3k1*^+*/-*^ altered the level of Krt 1 expression; however, their combination significantly increased Krt 1 in more than 20 samples examined (Fig. 5C). The in vivo and in vitro data together raise an intriguing possibility that the gene-environment interactions significantly potentiate basal to suprabasal differentiation as a potential mechanism underlying the eye developmental abnormalities.

## Discussion

In this paper, we describe an experimental system that differentiates mESCs to basal keratinocytes in vitro. The system enables convenient incorporation of genetic and environmental components, leading to the findings that *Map3k1* loss-of-function and dioxin, while do not affect mESC differentiation to surface ectoderm and basal keratinocytes, jointly potentiate basal to suprabasal epidermal differentiation. Compelled by these in vitro findings, we examined the gene-environment interactions in vivo and found that in utero dioxin exposure indeed increased Krt 1 expression more abundantly in the *Map3k1*^+*/-*^ than in the wild type embryos. These data suggest that the in vitro system described here can be used to explore complex conditions and etiology in the perturbation of epithelial differentiation.

Dioxin is a ubiquitous environmental agent that is stable and persistent in the environment and biological systems^45^. Consistent with the notion that most toxic effects of dioxin are mediated through the AHR, we found a good correlation between *Ahr* expression and dioxin effects on differentiation^46^. The minimal *Ahr* expression in mESCs and early phase of differentiation corresponded with unaltered differentiation from mESC to progenitor to basal keratinocytes in the presence of dioxin. The gradually increased *Ahr* and the steady-state high expression in the basal keratinocytes corresponded to potentiation of basal to spinous differentiation by dioxin. A similar observation has been made in the human cell culture models where dioxin is found to accelerate keratinocyte terminal differentiation, but does not change proliferation and apoptosis^25,26^. It is worth noting that of the many clinical manifestations of dioxin exposure, chloracne, a hyperkeratotic skin disorder is the most consistent pathology observed in exposed humans^47,48^. Thus, potentiation of basal to spinous differentiation observed here is likely relevant to dioxin-induced skin pathogenesis.

How dioxin accelerates differentiation remains unclear. Dioxin treatment of basal keratinocytes led to a significant up-regulation of *Cyp1a1*, an AHR-regulated detoxification gene. However, the detoxification gene products have not been causally linked to *Krt 1* expression and differentiation. The dioxin-AHR axis also regulates expression of genes that are not implicated in detoxification, such as transforming growth factor-a, epidermal growth factor^49^, Interleukin-1b and Plasminogen activator inhibitor-2^50^. Some of these gene products may mediate the toxicities of dioxin. For example, in a mouse model of embryonic palate fusion, Abbott et. al. showed that dioxin induces epithelial differentiation abnormalities to cause cleft palate in wild type, but not *Egf* knockout mouse palates, implicating a role for EGF in dioxin toxicity^51^.

We have previously shown that MAP3K1 is a signaling molecule crucial for eye development and that the *Map3k1*^*-/-*^ but not *Map3k1*^+*/-*^ embryos have eyelid closure defects due to epithelial morphogenetic abnormalities^16^. More recently, we found that *Map3k1* gene mutations sensitize the developmental programs to the toxicity of dioxin-like environmental chemicals^44^. Specifically, in utero dioxin exposure induces eye defects in *Map3k1*^+*/-*^ but not wild type embryos. The in vitro mESC epithelial differentiation model, followed by in vivo validation, suggests that the gene (*Map3k1*)-environment (dioxin) interactions affect epithelial differentiation. The in vitro and in vivo data present a coherent narrative that *Map3k1* mutation and dioxin have small effects on promoting basal to suprabasal differentiation, which is further potentiated by both agents together. The differentiation abnormalities induced by dioxin plus *Map3k1*^+*/-*^ resemble those in *Map3k1*^*-/-*^ cells, raising an intriguing possibility that the developmental defects occur when the differentiation abnormalities reaching beyond a threshold level.

Our differentiation protocol is modified from Metallo et. al. and Bilousova, et. al. with improved efficiency^32,33,52^. Using this protocol, we detected a gradual increase of *Krt 18* expression, reaching the peak level in 10 days that are comparable to the time frame required for mouse stem cells to commit to ectodermal lineage in vivo^53^. We further obtained Krt 14-expressing cells after culturing for approximately a month, though this time frame was much longer than that took in vivo for ectodermal to basal epithelial cell conversion*.* Given that the Krt 18-positive to Krt 14-positive conversion requires DNA methylation to turn off the *Krt 18* promotor and multiple extracellular signals and transcription factors to activate the *Krt 14* promoter, we speculate that these epigenetic and transcription machineries are less robust in vitro than in vivo^54–56^.

In vivo, the basal layer epidermis has low Ca^2+^ concentrations that support basal keratinocyte proliferation, whereas the suprabasal layers have high Ca^2+^ concentrations to promote terminal differentiation. Additionally, the three-dimensional (3D) in vivo microenvironment facilitate the intricate cell–matrix interactions and differentiation gene expression^57^. The in vitro conditions, i.e. monolayer culture and the DKSFM media containing 0.15 mM calcium similar to the Ca levels in the basal layer epidermis, on the other hand, seem to favor basal keratinocyte proliferation but prevent differentiation^58,59^. Preliminary characterization shows that the D-KC, while lacking stem cell markers, are enriched with epidermal progenitors, explaining the continuously growth and subculture capacities of the Krt 14-positive cells.

In summary, we have established an experimental model that differentiates mESCs to keratinocytes that recapitulates epithelial differentiation from E3.5 to E15.5. This system can be used as a convenient tool to trace epithelial differentiation; the resultant basal keratinocytes can be amplified and cultured for a long period of time, serving as resources for epithelial molecular biology research. Using this system, we show that *Map3k1* loss-of-function mutation and dioxin act jointly to potentiate epithelial spinous differentiation, unveiling a potential mechanism through which gene-environment interactions, but not each agent separately, cause developmental abnormalities. Understanding the complex etiology of diseases will help the development of preventive strategies.

## Materials and methods

### Reagents, antibodies and chemicals

Soybean Trypsin Inhibitor (17,075–029), TrypLE and Defined Keratinocyte SFM (DKSFM) were from Gibco. Dulbecco's Modification of Eagle's Medium (DMEM; 10–017-CV) was purchased from Corning. Collagen (Col) IV (354,233) was from BD Biosciences. ESGRO Leukemia Inhibitory Factor (LIF) (10^7 U/ml, ESG1107), retinoic acid (RA; 302–79-4) and Hoechst 33,342 (B2261) were from Sigma; recombinant mouse bone morphogenetic protein-4 (BMP4; 120-05ET) was from PEPROTECH. Dioxin, i.e. TCDD, was from AccuStandard and dissolved in dimethyl sulfoxide (DMSO; 67–68-5, Sigma). The rest cell culture media and reagents, and antibodies are listed in Supplementary Table s2 and s3.

### Mice, mESC culture and differentiation

The wild type and *Map3k1*^+*/-*^ mice, in utero dioxin exposure and the collection and process of embryos for immunostaining were described before^44^. The wild type, *Map3k1*^+*/-*^ and *Map3k1*^*-/-*^ mESCs were obtained from pregnant mice as described^60^. The mESCs were expanded and maintained in DMEM supplemented with 15% Knockout™ Serum Replacement (Gibco), LIF (10000x), 2 mM glutamine, 1% nonessential amino acids, 1 mM sodium pyruvate, 2-mercaptoehanol (Gibco, 1000x), 100 U/ml penicillin and 100 μg/ml streptomycin (Cytiva), in a humidified incubator with 5% CO_2_ at 37 °C. Protocol describing mouse experiments and procedures was approved by IACUC of the University of Cincinnati, and all experiments were performed in compliance with the ARRIVE guidelines and UC guidelines and regulations.

The step-wise differentiation of mESCs to the epithelial lineages followed protocols in Bilousova, et al.^32^ and Metallo, et al.^33^ with modifications. Briefly, on day 0, mESCs were trypsinized and resuspended in DMEM with 15% fetal bovine serum (FBS), known as embryoid body (EB) media; cells (5 × 10^4^ cells/25 µl) were placed as droplets on a Petri dish lid and incubated as “hanging drops” to enable the formation of EBs that contained cells of the three primitive germ layers^30^. On day 2, the EBs (about 100 in number) were collected and transferred to a 100 mm ColIV-coated tissue culture dish in EB medium plus 1 μM RA and 25 ng/ml BMP4, conditions that selectively induce surface ectoderm differentiation. On day 4, media were changed to DKSFM plus RA and BMP4 to promote epithelial lineage differentiation. On day 8, media were changed to DKSFM for keratinocyte amplification. On day 13, many cells with epithelial morphology were moving outward from the EB center. The clumps at the EB center was removed by vacuum aspiration; the remaining cells were detached with TrypLE, resuspended in DKSFM containing 10 mg/ml trypsin inhibitor and passaged to a new ColIV-coated dish, as passage (P)1. The passaged cells can be continuously passaged for at least 20 generations and storage in and recover from liquid N_2_. In some experiments, dioxin (10 nM) were included in the media at different phases of culture.

### RNA isolation, reverse transcription and quantitative polymerase chain reaction (qPCR)

Total RNA was isolated using PureLink RNA Mini Kit (12,183,025) and reverse transcription was performed using SuperScript IV reverse transcriptase (18,090,010; Invitrogen) following manufacture’s protocols. qPCR was carried out using PowerUp SYBR Green Master Mix (4,367,659, Applied Biosystems) and the signals were detected with an Agilent Technologies Stratagene Mx3000P PCR machine. The PCR reactions ran for 40 cycles under the appropriate parameters for each pair of primers and fluorescence values were used to construct the amplification curve. Specifically, at 95c for 10 s and 60c for 1 min. A dissociation curve was performed after amplification by gradual rise in temperature from 65 to 95 °C with fluorescence signal measurement every 0.5 °C The results were normalized using *Gapdh*; *ΔΔCt* were used to calculate fold change. Data represent results of triplicates of 2 or more experiments. The sequences of PCR primers are listed in Supplementary Table s4.

### Immunofluorescence, microscopic image and quantification

The embryonic tissue sections were processed and immunohistochemistry was done as described previously^16,61^. Briefly, the embryonic/fetal heads were fixed in 4% paraformaldehyde at 4 °C overnight. The tissues were embedded in Optimal Cutting Temperature compound and frozen. The entire eye was processed for coronal sections at 12 μM. Cells grown on ColIV-coated coverslips were fixed with 4% paraformaldehyde at 4 °C for 10 min, permeabilized with PBS plus 0.2% Triton, and subjected to immunofluorescent staining. Primary antibodies were diluted at 1:100 and secondary antibodies and nucleus staining reagents were dilute at 1:400. Immunofluorescence and bright field images were captured using a Zeiss Axio microscope. For immunostaining of the embryonic tissues, the imagens were analyzed using the ImageJ software (National Institutes of Health, Bethesda, MD, USA). The epithelial cell layers expressing distinctive E-cadherin were outlined and the mean intensity values of Krt1 staining were measured. Krt1 level in the images was determined after background subtraction.

### Global gene expression and pathway analyses

The wild-type and *Map3k1*^*-/-*^ primary mouse keratinocytes were subjected to high-density microarray hybridization; the differential gene expression was analyzed as reported before^17^ and are available at GSE201823. The data were re-analyzed through identifying the significantly differential expressed genes using the cut-off criteria: log2 fold change > 1 or < -1, False Discovery Rates (FDR) < 0.1, and intensity > 200, and the biological process enrichment using Metascape as previously described^62^. The datasets generated and/or analyzed during the current study are available.

### Statistical analyses

Means and standard deviations were calculated based on at least three independent experiments, and analyzed using student’s two-tailed *t*-test. *p, ^#^*p* < 0.05, **p < 0.01 and ***p, ^###^*p* < 0.001 were considered statistically significant.

## Supplementary Information

Supplementary Information.

## Data Availability

The datasets and cells generated during and/or analyzed during the current study are available from the corresponding author on reasonable request.
