# Supplementary material for: The combined effects of Map3k1 mutation and dioxin on differentiation of keratinocytes derived from mouse embryonic stem cells
Source: Sci Rep. 2022 Jul 7;12:11482. doi: 10.1038/s41598-022-15760-z (PMC9263165; doi:10.1038/s41598-022-15760-z)
Supplement: Supplementary file 1 — Supplementary Information. [file 41598_2022_15760_MOESM1_ESM.pdf]

The combined effects of *Map3k1* mutation and dioxin on differentiation of keratinocytes derived from mouse embryonic stem cells

Jingjing Wang, Bo Xiao, Eiki Kimura, Maureen Mongan and Ying Xia*

Department of Environmental and Public Health Sciences, University of Cincinnati, College of Medicine

*Correspondence should be addressed to:
Ying Xia, PhD

Department of Environmental and Public Health Sciences

University of Cincinnati, College of Medicine

Cincinnati, Ohio 45267-0056

USA

Phone: 513-558-0371

Email: ying.xia@uc.edu

Supplementary Information

Supplementary Table 1. Epithelial differentiation genes in *Map3k1*$^{-/-}$ vs Wild type keratinocytes

Clone	Name	Symbol	*Map3k1*$^{-/-}$ vs Wild type (Fold)
V00830	Keratin complex 1, acidic, gene 10	*Krt 1-10*	3.23
M34398	Loricrin	*Lor*	2.52
X91825	Small proline-rich protein 1B	*Sprr1b*	1.74
AJ005562	Small proline-rich protein 2D	*Sprr2d*	2.13
AJ005566	small proline-rich protein 2H	*Sprr2h*	3.22
NM_025984	small proline rich-like 3	*Sprrl3*	2.75
NM_027137	small proline rich-like 7	*Sprrl7*	2.66

Supplementary Table 2. Cell culture reagents

Name	Cata No	Company
Fetal bovine serum	S11150	Atlanta Biological
Knockout™ Serum	1082028	Gibco
L-glutamine	25-005-Cl	Corning
Non-essential amino acid	11140-050	Gibco
Sodium pyruvate	11360-070	Gibco
Penicillin-Streptomycin	SV30010	Cytiva
2-mercaptoehanol	21985023	Gibco
0.25% Trypsin	15050-057	Gibco

Supplementary Table 3. List of antibodies

Name	Host species	Antibody type	Cata No	Company
Keratin 18 (Krt 18)	Mouse	Monoclonal	MA5-12104	Thermo Fisher
Keratin 14 (Krt 14)	Mouse	Monoclonal	MA5-11599	Thermo Fisher
Keratin 1 (Krt 1)	Rabbit	Polyclonal	905602	BioLegend
Zonula occludens-1 (ZO-1)	Rabbit	Polyclonal	61-7300	Themo Fisher
E-Cadherin (E-Cad)	Mouse	Monoclonal	610181	Biosciences Discovery
Rabbit IgG Alexa Fluor 488	Goat	Polyclonal	A11034	Thermo Fisher
Mouse IgG Alexa Fluor 568	Goat	Polyclonal	A11004	Thermo Fisher

Supplementary Table 4. Primers sequences in the qPCR

Gene	Forward (5'-3')	Reversre (5'-3')
Gapdh	AACGACCCCTTCATTGACC	TGAAGACACCAGTAGACTCC
Krt 14	GAGCGGCAAGAGTGAGATTT	CTTTGGTCTCCTCCAGGTTATTC
Krt 1	TTCATCGACAAGGTGCGCTTCCTA	TGGTCACGAACTCATTCTCTGCGT
Krt8	CTGGTGGAGGACTTCAAGAATAA	ATGCTTCGTCCACATCCTTC
Krt15	ACATGCTGCTGGACATCAA	GGGATACTTCTCTGACACCAATAC
Krt 18	TGCAGCTGGAGACAGAAATC	ATCCACTTCCACAGTCAATCC
Flg	GATCAGGCTCAGGAGGAAGA	GAAACGATATACCTGGAGATGC
Inv	GAGAAGCAGCATCAGAAGCC	CTACTTCTCCTGCTGTGTCC
Lor	TGAGGAGACACTAGAATTGGG	AAGTAAGGTCACCGGGTTGC
Nanog	AGGGTCTGCTACTGAGATGCTCTG	CAACCACTGGTTTTTCTGCCACCG
Oct4	GGCGTTCTCTTTGGAAAGGTGTTC	CTCGAACCACATCCTTCTCT
Cyp1a1	TTTGAGAAGGGCCACATCCG	GATAGCAGTTGTGACTGTGTCA
Ahr	GGCCAAGAGCTTCTTTGAATG	TGCCAGTCTCTGATTGTGC
CD34	AAGGCTGGGTGAAGACCCTTA	TGAATGGCCGTTTCTGGAAGT
Lgr5	CCTACTCGAAGACTTACCCAGT	GCATTGGGGTGAATGATAGCA
P63	CCCCGCCTCTTTGCAAATCT	GGGTTTCTATGAAACGCTGGATG
Sca-1	AGGAGGCAGCAGTTATTGTGG	CGTTGACCTTAGTACCCAGGA

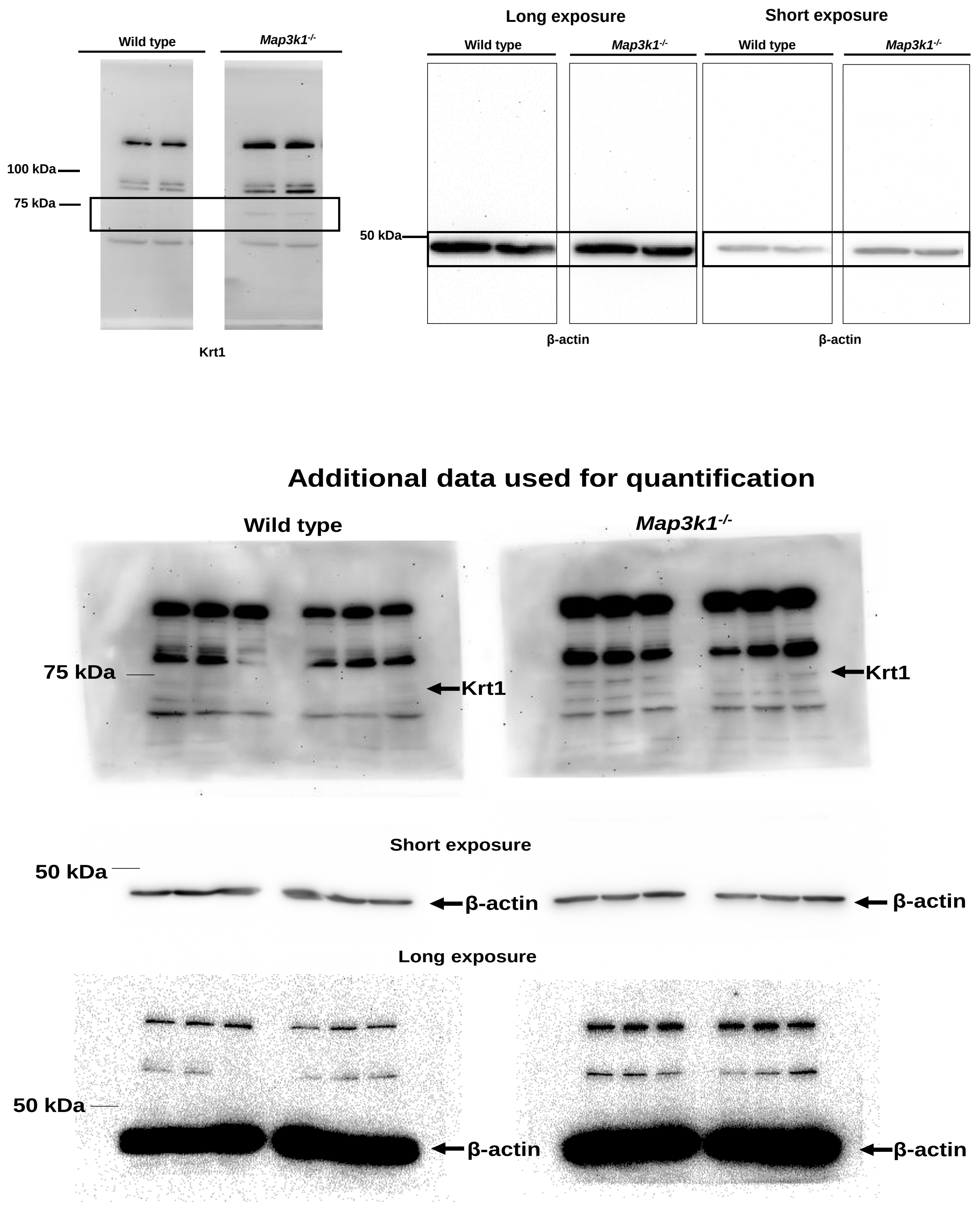

Supplementary Figure 1. Original blots of Krt1 and β-actin in wild type and *Map3k1$^{-/-}$* D-KC corresponding to Fig. 3D and data used for quantification corresponding to Fig. 3E
